# Psychosocial Factors Associated With Adherence to COVID-19 Preventive Measures in Low-Middle- Income Countries, December 2020 to February 2021

**DOI:** 10.3389/ijph.2022.1604398

**Published:** 2022-05-11

**Authors:** Supa Pengpid, Karl Peltzer, Chutarat Sathirapanya, Phanthanee Thitichai, Edlaine Faria de Moura Villela, Tamara Rodrigues Zanuzzi, Felipe de Andrade Bandeira, Suzanna A. Bono, Ching Sin Siau, Won Sun Chen, M Tasdik Hasan, Philippe Sessou, John D. Ditekemena, Mina C. Hosseinipour, Housseini Dolo, Rhoda K. Wanyenze, Joseph Nelson Siewe Fodjo, Robert Colebunders

**Affiliations:** ^1^ Department of Health Education and Behavioral Sciences, Faculty of Public Health Mahidol University, Ratchathewi, Thailand; ^2^ Department of Research Administration and Development, University of Limpopo, Polokwane, South Africa; ^3^ Department of Psychology, College of Medical and Health Science, Asia University, Taichung, Taiwan; ^4^ Department of Family and Preventive Medicine, Faculty of Medicine, Prince of Songkla University, Hat Yai, Thailand; ^5^ FETP Division, Department of Diseases Control, Ministry of Public Health, Nonthaburi, Thailand; ^6^ Disease Control Coordination, São Paulo State Health Department, São Paulo, Brazil; ^7^ Institute of Tropical Pathology and Public Health, Federal University of Goiás, Goiânia, Brazil; ^8^ School of Medicine, Health Sciences Unit, Federal University of Jataí, Jataí, Brazil; ^9^ School of Social Science, Universiti Sains Malaysia, Gelugor, Malaysia; ^10^ Centre for Community Health Studies (ReaCH), Faculty of Health Sciences, Universiti Kebangsaan Malaysia, Kuala Lumpur, Malaysia; ^11^ Department of Health Science and Biostatistics, Faculty of Health, Arts and Design, Swinburne University of Technology, Hawthorn, VIC, Australia; ^12^ Public Health Foundation, Bangladesh (PHF, BD), Dhaka, Bangladesh; ^13^ Department of Primary Care and Mental Health, University of Liverpool, Liverpool, United Kingdom; ^14^ Research Unit on Communicable Diseases, Polytechnic School of Abomey-Calavi, University of Abomey-Calavi, Cotonou, Benin; ^15^ Kinshasa School of Public Health, University of Kinshasa, Kinshasa, Democratic Republic of the Congo; ^16^ University of North Carolina UNC Project Malawi, Lilongwe, Malawi; ^17^ International Center of Excellence in Research, Faculty of Medicine and Odontostomatology, University of Sciences, Techniques and Technology of Bamako, Bamako, Mali; ^18^ Lymphatic Filariasis Research Unit, International Center of Excellence in Research, Faculty of Medicine and Odontostomatology, Centre Hospitalier Universitaire du Point-G, Bamako, Mali; ^19^ School of Public Health, College of Health Sciences, Makerere University, Kampala, Uganda; ^20^ Global Health Institute, University of Antwerp, Antwerp, Belgium

**Keywords:** psychosocial factors, low-and middle-income countries, COVID-19, adherence, preventive measures

## Abstract

**Objectives:** To investigate psychosocial factors associated with adherence to COVID-19 preventive measures in low- and middle-income countries (LMICs).

**Methods:** This online cross-sectional survey included 10,183 adults (median age 45 years) from nine LMICs. Participants were asked about adhering to four COVID-19 preventive measures (physical distancing, wearing a face mask, hand, and cough hygiene); a composite adherence score was calculated, ranging from 0–4 positive responses. Psychosocial measures included worry, anxiety, depression, social and demographic, and COVID-19 related factors.

**Results:** Factors associated with adherence to more preventive measures included being a participant from Malaysia or Bangladesh, older age, higher education, belonging to the healthcare sector (either as or worker), having health personnel as a trusted source of COVID-19 information/advice, possessing correct COVID-19 knowledge, worry or fear about being (re)infected with COVID-19, and screening negative for general anxiety symptoms.

**Conclusion:** Moderate to high adherence to COVID-19 preventive measures was found, with significant variations across countries. Psychosocial factors (worry, anxiety, knowledge, education, age, and country) seemed determinant in predicting the number of measures to which participants adhered.

## Introduction

COVID-19 preventive measures include wearing face masks, hand and cough hygiene, physical distance, and avoiding social gatherings [1]. In low- and middle-income countries (LMICs), for example, in Brazil in April 2020, hand hygiene was practiced by 98.7% of participants; 92.6% kept physical distancing, 94.2% adhered to cough hygiene, and 45.5% wore face masks [2], and in two studies in Mozambique in April and June 2020, the prevalence of wearing face masks ranged from 93.9% to 96.5%, likewise physical distancing ranged from 82.2% to 86.7%, regular hand washing 95.4%–96.4%, and covering mouth after coughing/sneezing 96.6%–96.9% [3]. In Malaysia in April–May 2020, 92.1% reported always wearing face masks in public places [4], and in Thailand in March 2020, 94.0% adhered to wearing face masks [5]. In Ecuador in November 2020, 92.6% of participants reported physical distancing, 93.4% hand hygiene, and 93.2% wore a face mask [6], in South Africa from April to May in 2020, 95.2% reported physical distancing, 95.4% hand hygiene, and 81.4% wore face masks [7], and in the Democratic Republic of Congo from April to June 2020, 41.7% were non-adherent to physical distancing and 15.3% were non-adherent to hand hygiene [8].

COVID-19 health protective behaviours may be based on an understanding of various sociocultural, cognitive, and psychosocial factors [9]. Sociocultural and health factors associated with adherence to COVID-19 preventive measures in low- and middle-income countries include sociodemographic factors, such as older age [2, 3, 10], gender/sex [2, 7, 10], being married [7, 10], higher education [2, 10], higher socioeconomic status [8, 10], living with other people [7], urban residence [2, 11, 12], and being a health care worker [2, 3]. Other factors associated with higher adherence to COVID-19 preventive measures include comorbidity [2], not having flu-like symptoms [7, 11], having flu-like symptoms [3], being tested for COVID-19 [9], obtaining COVID-19 information from a health care worker [3, 9, 12], correct COVID-19 knowledge [13], and concern about own health [12].

There is a lack of studies investigating the impact of mental problems on adherence to COVID-19 preventive measures [1]. A large study in 48 countries found that “the more stressed people felt, the less adherent they were with preventive COVID-19 behavioural guidelines; but also, that more concerned individuals tended to be more adherent with preventive measures.” [14]. Added to that, a study in Taiwan associated increased anxiety symptoms with higher scores of adherence to COVID-19 preventive measures [15], and in Slovenia, improved preventive behaviour towards COVID-19 was reported among persons who were more anxious and had higher psychological distress [16]. In a study in Cyprus, having depressive symptoms decreased adherence to COVID-19 preventive measures, while anxiety symptoms were positively correlated with “personal hygiene and indoors-related preventive measures.” [17]. In a large online survey among the general adult population in Germany early 2020, general anxiety symptoms were positively associated with adherence to COVID-19 safety behaviours [18]. During the severe acute respiratory syndrome (SARS) in 2003 in Hong Kong, among the general adult population higher anxiety levels were associated with higher uptake of protective measures [19], and in an online survey in June/July 2020 in Hong Kong, people with psychological distress were more likely to adhere to COVID-19 preventive measures [20]. Based on few previous studies in high-income countries, it can be hypothesized that psychological distress (anxiety, depression) influences adherence to COVID-19 preventive measures. So far, previous studies in low- and middle-income countries have focused on assessing adherence to COVID-19 preventive measures but without looking into its psychosocial determinants. We therefore conducted this multi-country survey among residents in low- and middle-income settings, with one of the main research objectives being the investigation of psychosocial factors in relation to adherence to COVID-19 preventive measures.

## Methods

### Study Design, Sample, and Procedure

This was a descriptive cross-sectional online study of adults in nine low- and middle-income countries between 10 December 2020 to 9 February 2021. This study was organised by the International Citizen Project (ICP) COVID-19 (ICPCovid) to monitor adherence to COVID-19 prevention in LMICs [21]. Study countries were selected based on their willingness to participate in the ICP; 50 participants per country was the minimum requirement for enough statistical power [14]. Participant inclusion criteria were 18 years and older, internet access and provision of informed consent. The study was approved by the Ethics Committees of all participating countries. Further details have been described previously [22]. Briefly, using nonprobability sampling as an online recruitment strategy, “questionnaires were completed online using an electronic link disseminated via the social network of the investigators, using platforms such as WhatsApp, Facebook, SMS, Messenger, Twitter, Instagram, and university webpage portals.” [22].

In the current study, we aimed to investigate psychosocial factors associated with adherence to COVID-19 preventive measures among adults in nine LMICs in different phases of the pandemic. Participating countries included Brazil from South America, Malaysia, Bangladesh, and Thailand from Southeast Asia, and the Democratic Republic of the Congo (DRC), Benin, Mali, Malawi, and Uganda from Africa. The five African countries were combined in the analysis since they had a low participation rate and were in a similar stage of the COVID-19 pandemic. On 31 January 2021, the daily new confirmed COVID-19 cases per million people were in Brazil 130.38, Malaysia 161.64, Thailand 11.85, Bangladesh 2.22, Malawi 23.72, Benin 8.59, Mali 1.05, Uganda 0.98, and DRC 1.81 [23]. In Brazil, physical distancing and confinement measures were implemented by the government in March 2000 [2] and mask wearing became mandatory in July 2000 [24]. However, the Brazilian population was also exposed to fake news, such as that “social isolation and use of facial masks are not efficient against the spread of COVID-19” [25]. In Malaysia from May 2020 the Conditional Movement Control Order (CMCO) comprised “wearing a face mask, washing hands frequently with hand sanitizer or soap and water, social distancing, and avoiding crowded places.” [26]. The DRC government implemented mandatory use of face mask and physical distancing since April 20, 2020 [8]. As from April 2020, the “Thai government’s recommendations have included the wearing of a face mask, practicing hand hygiene using alcohol gel, practicing food hygiene by not sharing eating utensils or drinking vessels, and physical distancing” [27], and in January 2021 COVID-19 “control measures were tightened and people were strongly admonished to wear masks, practice hand hygiene, and socially distance when outside the home” [28].

### Measures

The ICP consortium generated a core questionnaire in English [21], which was modified by the different country investigators, translated into the national languages of the study countries using standard scientific procedures of the participating countries, and questionnaires were pilot tested in all study countries [22]. In addition, some existing standardized questionnaires, such as the Patient Health Questionnaire (PHQ-2) and the Generalized Anxiety Disorder (GAD-2), were added, using study country language validated versions (where available).

### Outcome Variable

#### COVID-19 Preventive Measures

Four questions assessing preventive behaviours were retained for analysis. Participants were asked, “During the past 7 days, have you been observing any of the following preventive measures against COVID-19? 1) Physical distancing of at least 1.5 m 2) Wearing a face mask 3) Hand hygiene (regular handwashing with soap or using hand gel) and 4) Cough hygiene (covering the mouth when coughing or sneezing)”. Overall adherence was assessed by first coding each positive response as “1” while negative responses scored “0”, and then summing up the scores for the four measures as reported by each participant, thereby yielding a quantitative measure of adherence ranging from 0–4 (Cronbach’s alpha 0.7).

#### Covariates

Psychosocial measures included psychological variables, sociodemographic, and COVID-19 related factors.

*Psychological variables* were sourced from: 1) one Likert scale item assessing the *“*level of fear/worry of being infected with COVID-19” (ranging from 1 = not at all worried to 5 = extremely worried) [22]; 2) two items from the “Patient Health Questionnaire (PHQ-2)” on depression symptoms (depression considered if PHQ-2 score was ≥3) [29] (Cronbach alpha in this sample 0.75); and 3) two items from the “Generalized Anxiety Disorder (GAD-2)” tool for general anxiety symptoms (anxiety considered if GAD-2 score ≥3 scores) [30] (Cronbach alpha in this sample 0.82). The PHQ-2 and GAD-2 are reliable and valid screening tools for depressive and anxiety symptoms [29, 31].

*Sociodemographic variables* included education, number of housemates, sex, age, country, residential, and subjective socio-economic status. The latter was sourced from the question, “Which of the following categories best describes your current socio-economic situation? Low-income category, lower-middle income category, upper-middle income category, and high-income category.”

*COVID-19 related variables* included studying or working in the healthcare sector, most trusted source of COVID-19 information/advice (whereby the response categories “other”, including family and friends, “radio/TV”, “social media”, and “religious authorities” were all coded as “0”, and “health personnel” coded as 1); COVID-19 knowledge; Status of COVID-19 testing/infection status (1 = not tested/not knowing test results, 2 = tested negative, and 3 = tested positive); chronic/underlying diseases (diabetes, hypertension, heart disease, cancer, HIV, tuberculosis, and asthma; coded as “0” if none and “1” if at least one disease); having been quarantined (either at home or elsewhere) at any point in time during the COVID-19 epidemic. Correct COVID-19 knowledge was defined as all three affirmative responses to “1) if there is a possibility of being reinfected after recovering from a previous COVID-19 infection; 2) if COVID-19 infection could be prevented by a vaccine; and 3) if there is currently an effective vaccine against COVID-19.” [22]. Cronbach’s alpha for the COVID-19 knowledge measure was 0.7 in this study.

#### Data Management and Analyses

The completed survey questionnaires were exported from the secured server of the ICPCovid website and subjected to data cleaning and coding and transferred to STATA for analyses [8]. Having a representative sample of the population is essential particularly for comparative studies involving countries of different population size. Therefore, the country-wide weight, defined as the adjustment of the sample proportion in the representation of individual country population (aged at least 15 years in each country in 2019), was estimated for each country [32]. It is critical to correct any deviations of the “estimates and to compensate the effects on the estimates due to bias arising from over- and under-coverage.” [33, 34].

Descriptive statistics were used to describe the study population. Ordinal logistic regression was used to assess associations between sociodemographic factors, COVID-19 related factors, psychological factors, and the participant’s overall adherence to COVID-19 preventive measures (dependent variable), overall and for Brazil, Malaysia, Thailand and five African countries. Covariates with *p* < 0.1 in univariate analysis were subsequently included in the multivariable ordinal logistic regression model. Furthermore, multiple logistic regression was used to estimate associations between sociodemographic factors, COVID-19 related factors, psychological factors, and adherence to each of the four COVID-19 preventive measures as dependent variables (Physical distancing, wearing a face mask, hand, and cough hygiene). For these additional models, covariates with *p* < 0.05 were subsequently included in the multivariable logistic regression models. Statistical analyses were conducted using “STATA software version 15.0” (Stata Corporation, College Station, Texas, United States).

## Results

### Sample Characteristics

The sample included 10,183 adults (median age 45 years, interquartile range 33–57 years, range 18–93 years) from nine low- and middle-income countries (6,470 Brazil, 1738 Malaysia, 1,124 Thailand, 230 Bangladesh, 219 DR Congo, 159 Benin, 107 Uganda, 81 Malawi, and 55 Mali). Most participants (80.4%) resided in urban areas, 64.9% were female, 47.5% had a postgraduate education, and 34.4% were students or workers in health care. The most trusted source of COVID-19 information/advice was health care personnel (61.7%), 51.5% had correct COVID-19 knowledge, 29.2% had at least one chronic/underlying disease, and 38.2% had been quarantined during the COVID-19 epidemic. More than half of the participants (51.7%) were very or extremely worried about being (re)infected with COVID-19, 20.2% had depressive symptoms, and 21.7% had general anxiety symptoms. The highest adherence to COVID-19 preventive measures in the past 7 days was found for wearing a face mask (95.9%), followed by hand hygiene (88.7%), cough hygiene (66.9%), and physical distancing (66.8%). More than half of the participants (52.2%) adhered to all four COVID-19 preventive measures (Table 1).

**TABLE 1:** Sample and COVID-19 preventive measures characteristics of adults in nine low-and middle-income countries, 2020, 2021.

Variable	Sample	Physical distancing	Wearing face mask	Hand hygiene	Coughing hygiene	Composite adherence score				
						0	1	2	3	4
	N (%) or M (SD)	%	%	%	%	%	%	%	%	%
Sociodemographic factors										
All	10,183	66.8	95.9	88.7	66.9	2.2	7.1	13.1	25.5	52.2
Country (% student/worker in health care)										
Brazil (30.4%)	6,470 (63.5)	64.3	95.3	88.6	64.2	2.9	7.3	14.7	24.7	50.4
Malaysia (21.3%)	1,738 (17.1)	88.4	97.8	92.5	76.5	1.1	3.5	4.9	20.4	70.1
Thailand (55.0%)	1,124 (11.0)	59.1	97.8	86.8	66.6	0.5	8.1	15.2	32.8	43.3
Bangladesh (57.8%)	230 (2.3)	51.3	95.7	89.1	80.0	1.3	8.3	7.8	38.3	44.3
5 African countries (66.7%)	621 (6.1)	52.8	93.9	82.1	63.9	1.1	12.9	16.9	30.3	38.8
Age in years										
18–34	2,913 (28.6)	56.3	95.2	84.7	66.5	2.3	9.8	15.5	27.6	44.8
35–49	3,017 (29.8)	63.7	95.7	87.1	63.3	2.5	8.1	15.7	24.5	49.2
50 or more	4,233 (41.6)	76.3	96.6	92.5	69.8	1.9	4.5	9.6	24.8	59.3
Sex										
Male	3,579 (35.1)	68.5	95.4	87.8	66.6	2.1	7.4	12.9	25.3	52.2
Female	6,604 (64.9)	65.9	96.2	89.1	67.1	2.3	6.9	13.2	25.6	52.1
Living status										
Lives alone	932 (9.2)	70.4	95.4	89.7	67.6	2.5	5.7	10.8	28.3	52.7
Lives with other people	9,251 (90.8)	66.5	96.0	88.6	66.8	2.2	7.2	13.3	25.2	52.1
Education										
Primary/secondary	1,316 (13.0)	62.8	93.0	83.4	63.8	3.4	9.9	14.5	24.6	47.6
Undergraduate	4,028 (39.5)	64.1	95.8	88.1	67.0	2.2	7.6	13.1	27.3	49.9
Postgraduate	4,839 (47.5)	70.3	96.9	90.6	67.7	1.9	5.9	12.6	24.2	55.4
Residence										
Rural	812 (8.0)	68.5	96.3	85.1	68.1	1.1	9.9	11.1	25.9	52.1
Suburban/urban slum	1,185 (11.6)	62.6	95.0	86.7	66.0	2.7	8.8	13.8	24.9	49.8
Urban	8,186 (80.4)	67.3	96.0	89.3	66.9	2.2	6.5	13.2	25.5	52.5
Income										
Low/lower middle	5,298 (52.0)	62.8	95.2	86.6	65.2	2.5	8.5	14.1	26.3	48.6
Upper middle/high	4,885 (48.0)	71.2	96.7	90.9	68.7	1.8	5.5	12.0	24.6	56.0
COVID-19 related factors										
Student or worker in the healthcare sector	3,500 (34.4)	62.5	95.9	88.1	68.6	2.0	7.9	13.2	26.7	50.1
Most trusted source of COVID-19 information/advice										
Other	3,905 (38.3)	66.7	95.1	86.9	64.4	2.4	8.2	13.7	25.6	50.2
Health care personnel	6,278 (61.7)	66.9	96.5	89.8	68.4	2.1	6.4	12.7	25.4	53.4
COVID-19 correct knowledge	5,145 (51.5)	69.8	96.5	90.1	67.7	2.3	6.2	12.0	24.3	55.3
COVID-19 testing										
Not tested/not knowing results	6,078 (59.7)	68.6	96.2	88.8	67.3	1.8	6.9	12.4	26.2	52.6
Negative	3,362 (33.0)	66.6	96.0	89.4	66.9	2.5	6.9	13.3	24.1	53.3
Positive	743 (7.3)	53.7	93.8	84.1	63.1	3.9	9.3	18.0	25.7	43.1
Chronic condition(s)	2,974 (29.2)	72.7	96.0	90.2	66.1	2.4	5.6	11.9	24.9	55.2
Has been quarantined during COVID-19 epidemic	3,889 (38.2)	64.2	95.3	87.9	65.5	2.8	7.6	14.3	24.3	50.9
Psychological factors										
Worry/fear about being (re) infected with COVID-19 (very or extremely)	5,160 (51.7)	70.6	95.7	88.4	66.5	3.0	6.7	12.1	22.5	55.7
Depressive symptoms	2,058 (20.2)	65.2	94.3	86.8	65.1	3.5	8.0	13.8	22.9	51.7
General anxiety symptoms	2,212 (21.7)	61.0	94.4	84.9	62.1	4.0	9.1	15.4	23.4	48.1

### Adherence to Composite COVID-19 Preventive Measure

In adjusted ordinal logistic regression, worry or fear about being (re)infected with COVID-19 increased the odds (1.14) of adhering to more preventive measures, general anxiety symptoms decreased them (0.73), and depressive symptoms did not significantly decrease adhering to more preventive measures. The odds of adhering to more COVID-19 preventive measures increased from 1.20 (in the 35–49 years age group) to 1.84 (in the ≥50 years age group) when compared to the younger people aged 18–34 years. Furthermore, these odds increased from 1.27 among undergraduates to 1.47 among postgraduates when compared to participants with only primary or secondary education. Students or workers in the healthcare sector had higher odds (1.28) to adhere to more preventive measures than other respondents, those that had correct COVID-19 knowledge, and those whose trusted source of COVID-19 information was health personnel were also more likely to adhere to more measures. Participants from Malaysia and Bangladesh had higher odds (3.91, and 1.74, respectively) to comply with more preventive measures than participants from Brazil (Table 2).

**TABLE 2:** Ordinal logistic regression with adherence to COVID-19 preventive measures in nine low-and middle-income countries, 2020, 2021.

Variable	COR (95% CI)	*p*-value	AOR (95% CI)	*p*-value
Sociodemographic factors				
Country				
Brazil	1 (References)	—	1 (References)	—
Malaysia	2.52 (2.25, 2.82)	<0.001	3.91 (3.34, 4.57)	<0.001
Thailand	0.86 (0.77, 0.96)	0.008	1.02 (0.86, 1.21)	0.827
Bangladesh	0.96 (0.77, 1.20)	0.708	1.74 (1.34, 2.28)	<0.001
5 African countries	0.66 (0.56, 0.77)	<0.001	1.03 (0.84, 1.27)	0.745
Age in years				
18–34	1 (References)	—	1 (References)	—
35–49	1.16 (0.98, 1.37)	0.078	1.20 (1.01, 1.44)	0.044
50 or more	1.78 (1.54, 2.06)	<0.001	1.84 (1.56, 2.18)	<0.001
Female (base = male)	1.13 (0.97, 1.30)	0.107	—	—
Living status				
Lives alone	1 (References)	—	—	—
Lives with other people	0.88 (0.74, 1.05)	0.147	—	—
Education				
Primary/secondary	1 (References)	—	1 (References)	—
Undergraduate	1.21 (0.96, 1.52)	0.106	1.27 (0.99, 1.63)	0.058
Postgraduate	1.63 (1.29, 2.06)	<0.001	1.47 (1.14, 1.89)	0.003
Residence				
Rural	1 (References)	—	—	—
Suburban/urban slum	1.16 (0.89, 1.51)	0.277		
Urban	1.09 (0.88, 1.36)	0.418		
Income				
Low/lower middle	1 (References)	—	1 (References)	—
Upper middle/high	1.42 (1.23, 1.63)	<0.001	1.16 (0.99, 1.35)	0.073
COVID-19 related factors				
Student or worker in the healthcare sector	1.15 (1.00, 1.32)	0.054	1.28 (1.09, 1.50)	0.002
Most trusted source of COVID-19 information/advice				
Other	1 (References)	—	1 (References)	—
Health care personnel	1.43 (1.24, 1.65)	<0.001	1.39 (1.20, 1.62)	<0.001
COVID-19 correct knowledge	1.53 (1.34, 1.75)	<0.001	1.43 (1.23, 1.67)	<0.001
COVID-19 testing				
Not tested/not knowing results	1 (References)	—	1 (References)	—
Negative	1.15 (0.99, 1.34)	0.072	1.03 (0.88, 1.22)	0.689
Positive	0.95 (0.70, 1.29)	0.749	0.84 (0.61, 1.15)	0.268
Chronic condition(s)	1.26 (1.08, 1.47)	0.004	1.00 (0.84, 1.18)	0.975
Has been quarantined during COVID-19 epidemic	1.12 (0.96, 1.30)	0.139	—	—
Psychological factors				
Worry/fear about being (re)infected with COVID-19	1.19 (1.12, 1.26)	<0.001	1.14 (1.06, 1.21)	<0.001
Depressive symptoms	0.86 (0.71, 1.03)	0.100	—	—
General anxiety symptoms	0.79 (0.66, 0.94)	0.008	0.73 (0.60, 0.89)	<0.001

COR, Crude Odds Ratio; AOR, Adjusted Odds Ratio; CI, Confidence Interval.

Furthermore, while worry or fear about being (re)infected with COVID-19 increased the odds of adhering to more preventive measures in Brazil, Malaysia, and five African countries, general anxiety symptoms decreased them in Brazil only, and depressive symptoms did not decrease the odds of adhering to more preventive measures in any of the study countries. The odds of adhering to more COVID-19 preventive measures increased by age group in Brazil, Malaysia, and Thailand, but not in five African countries. Living with other people increased the odds of adhering to more COVID-19 preventive measures in Malaysia. The odds of adhering to more COVID-19 preventive measures increased by higher education in Brazil and Thailand, but not in Malaysia and in five African countries. Participants with higher income adhered to more COVID-19 adherence measures in Brazil but not in Malaysia, Thailand, and five African countries. Students or workers in the healthcare sector had higher odds to adhere to more preventive measures than other respondents in Thailand and in five African countries but not in Brazil and Malaysia. For those whose trusted source of COVID-19 information was health personnel were also more likely to adhere to more measures in Brazil, Thailand, and in five African countries, but not in Malaysia. Correct COVID-19 knowledge increased the odds of more preventive measures in Brazil, but not in Malaysia, Thailand, and five African countries. Participants who had tested positive for COVID-19 had lower odds in Brazil and Malaysia and higher odds in five African countries of adhering to more preventive measures. Those that had quarantined during the COVID-19 epidemic in Thailand had higher odds of adhering to more preventive measures, while this was not the case in Brazil, Malaysia, and in five African countries (Table 3).

**TABLE 3:** Ordinal logistic regression with adherence to COVID-19 preventive measures in Brazil, Malaysia, Thailand and five African countries, 2020, 2021.

Variable	Brazil		Malaysia		Thailand		5 african countries	
	COR (95% CI)	*p*-value	AOR (95% CI)	*p*-value	AOR (95% CI)	*p*-value	AOR (95% CI)	*p*-value
Sociodemographic factors								
Age in years								
18–34	1 (References)	—	1 (References)	—	1 (References)	—	1 (References)	—
35–49	1.12 (0.97, 1.28)	0.111	1.82 (1.36, 2.44)	<0.001	1.50 (1.10, 2.07)	0.010	1.25 (0.88, 1.77)	0.212
50 or more	2.04 (1.77, 2.34)	<0.001	1.83 (1.37, 2.43)	<0.001	1.58 (1.17, 2.13)	0.003	0.97 (0.51, 1.82)	0.914
Female (base = male)	—	—	—	—	—	—	—	—
Living status								
Lives alone	1 (References)	0.574	1 (References)	0.013	—	—	—	—
Lives with other people	0.96 (0.83, 1.05)		1.68 (1.11, 2.52)					
Education								
Primary/secondary	1 (References)	—	1 (References)	—	1 (References)	—	—	—
Undergraduate	1.15 (0.98, 1.35)	0.080	0.84 (0.61, 1.14)	0.264	2.46 (1.70, 3.54)	<0.001		
Postgraduate	1.39 (1.18, 1.63)	<0.001	0.88 (0.63, 1.25)	0.482	2.89 (1.88, 4.42)	<0.001		
Residence	—	—	—	—	—	—	—	—
Rural								
Suburban/urban slum								
Urban								
Income								
Low/lower middle	1 (References)	<0.001	—	—	—	—	—	—
Upper middle/high	1.27 (1.15, 1.40)							
COVID-19 related factors								
Student/worker in healthcare	0.92 (0.83, 1.03)	0.140	—	—	1.86 (1.46, 2.37)	<0.001	1.41 (1.00, 1.96)	0.048
Most trusted source of COVID-19 information/advice								
Other	1 (References)	0.002	1 (References)	0.067	1 (References)	0.029	1 (References)	0.011
Health care personnel	1.17 (1.06, 1.29)		1.22 (0.99, 1.50)		1.34 (1.03, 1.74)		1.50 (1.10, 2.04)	
COVID-19 correct knowledge	1.56 (1.41, 1.72)	<0.001	—	—	—	—	1.40 (0.99, 1.97)	0.058
COVID-19 testing								
Not tested/not knowing results	1 (References)	—	1 (References)	—	—	—	1 (References)	—
Negative	0.93 (0.84, 1.04)	0.196	1.00 (0.77, 1.29)	0.996			1.21 (0.87, 1.68)	0.269
Positive	0.70 (0.60, 0.82)	<0.001	0.85 (0.49, 0.95)	<0.001			3.23 (1.10, 2.04)	0.033
Chronic condition(s)	0.98 (0.88, 1.09)	0.698	—	—	—	—	—	—
Has been quarantined during COVID-19 epidemic	0.97 (0.88, 1.07)	0.556	1.30 (0.96, 1.76)	0.085	1.43 (1.16, 2.81)	0.009	—	—
Psychological factors								
Worry/fear about being (re)infected with COVID-19	1.12 (1.06, 1.17)	<0.001	1.17 (1.05, 1.29)	0.003	—	—	1.27 (1.11, 1.46)	<0.001
Depressive symptoms	0.99 (0.86, 1.13)	0.831	—	—	—	—	—	—
General anxiety symptoms	0.82 (0.72, 0.93)	0.003	—	—	—	—	—	—

COR, Crude Odds Ratio; AOR, Adjusted Odds Ratio; CI, Confidence Interval.

### Adherence to Four Specific COVID-19 Preventive Measures

In adjusted logistic regression analysis, worry/fear about being (re)infected with COVID-19 were positively associated with adherence to physical distancing (AOR: 1.10, 95% CI: 1.02–1.19), while general anxiety symptoms were negatively associated with adherence to physical distancing (AOR: 0.74, 95% CI: 0.58–0.96) and hand hygiene (AOR: 0.63, 95% CI: 0.47–0.84). Depressive symptoms were not significantly associated with any of the four specific COVID-19 preventive measures. Participants from Malaysia (AOR: 6.39, 95% CI: 5.28–7.73), older age (≥50 years) (AOR: 2.04; 95% CI: 1.68–2.46), postgraduate degree (AOR: 1.43, 95% CI: 1.08–1.91), living in an upper middle- or high-income country (AOR: 1.24, 95% CI: 1.03–1.48), having health-care personnel as the most trusted source of COVID-19 information/advice (AOR: 1.20, 95% CI: 1.00–1.43), and correct COVID-19 knowledge (AOR: 1.46, 95% CI: 1.22–1.75) were positively associated with adherence to physical distancing. Compared to participants from Brazil, participants from Malaysia had higher odds of adhering to all four COVID-19 preventive measures, Thailand had higher odds of adhering to wearing face masks, Bangladesh had higher odds of adhering to cough hygiene, and participants from five African countries had lower odds of adhering to hand hygiene. Older age was associated with adhering to three preventive measures, except for wearing face masks. Higher education was associated with adhering to three COVID-19 prevention measures, except for cough hygiene. Participants from suburban or urban slums and/or urban areas were more likely to adhere to hand and cough hygiene than rural dwellers. Being a student or staff in health care was positively associated with adhering to cough hygiene only. Having health-care personnel as the most trusted source of COVID-19 information/advice was positively associated with adhering to all four preventive behaviours. Correct COVID-19 knowledge increased the odds of adhering to two preventive measures (physical distancing and hand hygiene) (Table 4).

**TABLE 4:** Logistic regression with individual adherence to COVID-19 preventive measures in nine low-and middle-income countries, 2020,2021.

Variable	Physical distancing of at least 1.5 m	Wearing face mask	Hand hygiene (regular handwashing with soap or using hand gel)	Cough hygiene (covering mouth when coughing or sneezing)
	AOR (95% CI) (*p*-value)	AOR (95% CI) (*p*-value)	AOR (95% CI) (*p*-value)	AOR (95% CI) (*p*-value)
Sociodemographic factors				
Country				
Brazil	1 (References)	1 (References)	1 (References)	1 (References)
Malaysia	6.39 (5.28, 7.73) (<0.001)	2.82 (1.90, 4.19) (<0.001)	1.86 (1.45, 2.39) (<0.001)	2.12 (1.83, 2.45) (<0.001)
Thailand	—	2.15 (1.37, 3.34) (<0.001)	—	—
Bangladesh	—	—	—	2.34 (1.66, 3.30) (<0.001)
5 African countries	—	—	0.67 (0.50, 0.91) (0.010)	—
Age in years				
18–34	1 (References)	1 (References)	1 (References)	1 (References)
35–49	1.30 (1.06, 1.59) (0.010)	0.84 (0.55, 1.29) (0.427)	1.01 (0.78, 1.29) (0.008)	0.91 (0.75, 1.10) (0.329)
50 or more	2.04 (1.68, 2.46) (<0.001)	1.12 (0.77, 1.62) (0.551)	1.51 (1.18, 1.94) (0.023)	1.20 (1.01, 1.42) (0.041)
Female (base = male)	—	—	1.24 (0.96, 1.59) (0.095)	—
Living status				
Lives alone	1 (References)	—	—	—
Lives with other people	0.88 (0.71, 1.10) (0.271)			
Education				
Primary/secondary	1 (References)	1 (References)	1 (References)	—
Undergraduate	1.17 (0.88, 1.55) (0.287)	2.10 (1.26, 3.56) (0.005)	1.56 (1.12, 2.17) (0.008)	
Postgraduate	1.43 (1.08, 1.91) (0.014)	3.11 (1.82, 5.33) (<0.001)	1.91 (1.35, 2.69) (<0.001)	
Residence				
Rural	—	—	1 (References)	1 (References)
Suburban/urban slum			1.83 (1.20, 2.80) [0.005]	1.43 (1.04, 1.96) [0.028]
Urban			1.46 (1.05, 2.02) [0.023]	1.06 (0.81, 1.38) [0.675]
Income				
Low/lower middle	1 (References)	—	—	1 (References)
Upper middle/high	1.24 (1.03, 1.48) [0.024]			1.11 (0.94, 1.32) [0.214]
COVID-19 related factors				
Student or worker in the healthcare sector	—	—	—	1.45 (1.22, 1.72) [<0.001]
Most trusted source of COVID-19 information/advice				
Other	1 (References)	1 (References)	1 (References)	1 (References)
Health care personnel	1.20 (1.00, 1.43) (0.045)	1.65 (1.15, 2.39) (0.007)	1.45 (1.15, 1.84) (0.002)	1.50 (1.28, 1.75) (<0.001)
COVID-19 correct knowledge	1.46 (1.22, 1.75) (<0.001)	—	1.40 (1.10, 1.77) (0.005)	—
COVID-19 testing				
Not tested/not knowing results	—	—	1 (References)	—
Negative			1.26 (0.97, 1.63) (0.085)	
Positive			0.81 (0.52, 1.26) (0.344)	
Chronic condition(s)	1.17 (0.95, 1.43) (0.141)	—	—	—
Has been quarantined during COVID-19 epidemic	—	—	—	—
Psychological factors				
Worry/fear about being (re)infected with COVID-19	1.10 (1.02, 1.19) (0.011)	—	1.02 (0.93, 1.13) (0.631)	—
Depressive symptoms	0.88 (0.67, 1.15) (0.339)	0.75 (0.46, 1.22) (0.246)	—	—
General anxiety symptoms	0.74 (0.58, 0.96) (0.023)	0.82 (0.52, 1.29) (0.382)	0.63 (0.47, 0.84) (0.002)	—

AOR, Adjusted Odds Ratio (including variables found significant <0.05 in univariate analysis), CI, Confidence Interval.

## Discussion

The study found that in nine low- and middle-income countries between December 2020 to February 2021, persons with greater worry/fear about being (re)infected with COVID-19 were more likely to adhere to more preventive measures, contrasting with persons with general anxiety symptoms who tended to observe fewer COVID-19 preventive measures. The increase of COVID-19 preventive behaviour with an increase of worry or fear about being (re)infected with COVID-19 seems to concur with previous research on health risk perception or health concerns [1, 12, 14]. Despite a possible detrimental health impact of excessive worry, this may help to stimulate COVID-19 preventive behaviours [15], in particular physical distancing. Like what has been reported in certain other studies [14, 17], general anxiety symptoms were negatively associated with adherence to COVID-19 preventive behaviour, in particular in Brazil. However, in studies in Taiwan and Slovenia, increased anxiety symptoms were significantly associated with increased preventive measures scores [15, 16], and in a study in Cyprus “personal hygiene and indoors-related precautionary measures” was positively correlated with anxiety symptoms [17]. In particular, in our study increased general anxiety symptoms were negatively associated with adherence to social distancing and hand hygiene behaviour. Similarly, the Cyprus study found an inverse correlation between “personal hygiene and indoors-related precautionary measures” and depressive symptoms [17]. It is possible that in the Taiwan study conducted in February 2020, at the beginning of the epidemic general anxiety levels were higher (48.8%) leading to higher adherence behaviour [15], while in our study later in the epidemic (December 2020-February 2021) general anxiety levels were lower (21.7%) associated with lower adherence behaviour. Unlike some previous research that showed that having depressive symptoms decreased adherence to COVID-19 preventive measures [17], and having psychological distress (anxiety, depression) increased adherence to COVID-19 preventive measures [20], we did not find any significant association between depressive symptoms and any specific and summative COVID-19 preventive behaviour. In high-income countries most studies show a positive association between anxiety and adherence to COVID-19 preventive measures, while depressive symptoms had negative or positive associations with adherence to COVID-19 preventive measures. In contrast to these findings, our study in LMICs found a negative association between anxiety and adherence to COVID-19 preventive measures, and no association between depressive symptoms and adherence to COVID-19 preventive measures. Based on cross-sectional studies it is impossible to determine whether general anxiety is a risk factor for non-adherence to preventive measures or whether it is the consequence of not being able to adhere to such measures, due to a lack of access to face masks and opportunities to wash hands and to respect physical distance because working at home is not an option. The latter could explain the observed differences concerning this association between high and LMICs.

Considering the four preventive measures assessed, the highest adherence was found for wearing a face mask, followed by hand hygiene, cough hygiene, and physical distancing; of note, about half of the respondents reported adhering to all four preventive measures but this proportion varied across study areas. Compared to a survey in April 2020 [2], in Brazil, social distancing reduced from 92.6% in 2020 to 64.3% as reported in this study, hand hygiene reduced from to 98.7%–88.7%, and coughing hygiene reduced from 94.2% to 66.9%, but wearing face masks increased from 45.5% in the 2020 Brazil survey to 95.3% in the Brazil December-2020/February 2021 survey. Compared to a survey in Mozambique in April and June 2020 [3], in this study of five African countries the prevalence of wearing a face mask (93.9%) was similar to the Mozambique survey (93.9%–96.5%), social distancing (52.8%) was lower than in the Mozambique survey (82.2%–86.7%), hand hygiene (82.1%) was also lower (95.4%–96.4%), and coughing hygiene (63.9%) was also lower than in the Mozambique survey (96.6%–96.9%). Compared to a previous survey in Malaysia in April–May 2020 [4], prevalence of wearing face mask (92.1%) increased to 97.8% in this survey in Malaysia and compared to a survey in Thailand in March 2020 [5], the prevalence of wearing face mask (94.0%) increased to 97.8% in this survey in Thailand.

The study found that sociodemographic factors (country, Malaysia, Bangladesh; older age and higher education) increased the odds for adhering to more COVID-19 preventive measures. The increased adherence with increasing age and higher education is consistent with previous research [1–3, 8, 10]. As older age is an important risk factor for the severity of COVID-19 illness, it is important to protect older adults from getting infected with COVID-19. Target groups to improve overall adherence to the preventive measures should therefore include younger and less educated individuals, particularly in Brazil, Thailand, and the five African countries. In contrast to some previous studies [7, 8, 10], which reported differences in adherence to COVID-19 preventive measures across genders and living status, our study did not find such disparities. However, in a country-stratified analysis, we found that living with other people in Malaysia increased the odds to adherence to more preventive measures. This result is consistent with a study in South Africa [7].

Other studies found an association between urban residence and adhering to COVID-19 preventive measures [2, 12, 13], while this study did not find a difference regarding residence status for the composite adherence measure. However, in this study participants from suburban or urban slum and/or urban areas were more likely to adhere to hand and cough hygiene than rural dwellers. People in rural areas may have perceived themselves at lower risk of contracting COVID-19 and may have less access to hand sanitizers or soap, making them less likely to observe strong hand hygiene; emphasis should be laid on such points when planning for communication campaigns targeting rural areas [2].

Regarding COVID-19 related factors, this study found that being a healthcare student or staff, having healthcare personnel as the most trusted source of COVID-19 information/advice, and possessing the correct COVID-19 knowledge increased the odds to adhere to more COVID-19 preventive measures. These findings are consistent with previous research results [2, 3, 10, 12]. Being a student or staff in the health care sector may be related to good preventive behaviour, because of them having been trained on the importance of adhering to COVID preventive measures [8]. As a result of the positive association between health care personnel as the most trusted source of COVID-19 information/advice and adherence to COVID-19 preventive measures, it may be important to governments to disseminate evidence-based messages to the public about COVID-19 preventive measures through these health-care personnel. Furthermore, the study found that participants who had tested positive for COVID-19 had lower odds in Brazil and Malaysia and higher odds in five African countries of adhering to more preventive measures, and in Thailand having quarantined increased the odds of more preventive measures. The higher odds of participants from five African countries and from Thailand adhering to more prevention measures may be attributed to the high proportion of students or workers in health care from these countries (Thailand 55.0% and 5 African countries 66.7%).

### Study Limitations

Although a large sample of the adult population participated in this survey, there was a selection bias towards including persons who had access to the internet and smartphones and had higher education. The method of distribution of the study tool through social media may have biased the sample towards an overrepresentation of students/workers in health care and with a higher educational level. Moreover, the fact that our study participants were not representative of the diversity in sociocultural and economic characteristics of these countries makes it difficult to carry out comparisons between countries. Since the study design was cross-sectional, we cannot determine causality for the found associations. Furthermore, the study was self-reported, which may have biased some responses. Our adherence measure used only a “Yes/No” format and only had four questions, while the use Likert scale questions and a higher number of questions could have led to more nuanced responses.

### Practice and Policy Implications

Generally, adherence to COVID-19 preventive measures was sub-optimal and decreased in some countries over time and need to be reinforced to reduce transmission risk. In particular, target groups to improve overall adherence to the preventive measures should include younger and less educated individuals, particularly in Brazil, Thailand, and the five African countries. Since study participants from rural areas were less likely to adhere to hand and cough hygiene than urban dwellers, rural dwellers may be targeted in communication campaigns for improving access to hand sanitizers or soap to observe hand hygiene and cough hygiene. Health care personnel were perceived as the most trusted source of COVID-19 information/advice, which could be utilized by governments to disseminate evidence-based messages to the public about COVID-19 preventive measures through these health-care personnel. The finding that participants who had tested positive for COVID-19 had lower odds in Brazil and Malaysia of adhering to more preventive measures, should lead to increased health education on the practice of COVID-19 preventive measures among those who had tested positive in Brazil and Malaysia. The results that an increase of COVID-19 preventive behaviour occurred with an increase of worry or fear about being (re)infected with COVID-19 may be used in sensitively framing information dissemination messages to balance negative and fear-based appeals and messages with positive and emotion-based messages that can lead to better appeals. Anxiety symptoms were found to be a barrier to COVID-19 preventive measures, to decreased adherence to social distancing and hand hygiene behaviour, and in Brazil, interventions should be directed at people with anxiety symptoms to improve adherence to COVID-19 preventive behaviours.

### Conclusion

In this survey conducted in nine low- and middle-income countries, a high adherence to wearing face masks and hand hygiene was observed, but only two-thirds of participants reported observing cough hygiene and physical distancing. Factors associated with adherence to more preventive measures included being a participant from Malaysia or Bangladesh, older age, higher education, belonging to the healthcare sector (either as or worker), having health personnel as a trusted source of COVID-19 information/advice, possessing correct COVID-19 knowledge, worry or fear about being (re)infected with COVID-19, and screening negative for general anxiety symptoms. These findings should be considered in designing population programmes to improve adherence to COVID-19 preventive measures in low resourced settings. Longitudinal and repeat surveys are needed to further monitor and evaluate the prevalence and potential predictors of adherence to COVID-19 preventive measures in the study and other countries considering the permanently changing COVID-19 situation globally.
